# Metabolomics for Athlete Monitoring: An Evidence-Readiness Framework for Contextual Validation

**DOI:** 10.3390/metabo16100704

**Published:** 2026-09-23

**Authors:** Donghai Lin, Yifen Chen, Caihua Huang, Zhiyun Zou

**Affiliations:** 1 Key Laboratory for Chemical Biology of Fujian Province, High-Field NMR Research Center, College of Chemistry and Chemical Engineering, Xiamen University, Xiamen 361005, China; 2 Research and Communication Center of Exercise and Health, Xiamen University of Technology, Xiamen 361024, China

**Keywords:** athlete monitoring, sports metabolomics, longitudinal metabolomics, biomarker panels, training adaptation, recovery

## Abstract

Metabolomics can place the metabolic consequences of training, competition, recovery, nutrition, and environmental exposure in a physiological context. However, an exercise-responsive metabolite should not be regarded as decision-ready on the basis of a pre–post association alone, because interpretation depends on the biological matrix, sampling time, analytical performance, athlete characteristics, and intended monitoring question. This structured narrative review examines the requirements for translating metabolomic findings into athlete monitoring. English-language, peer-reviewed studies were identified through structured searches of PubMed, Scopus, and Web of Science from 1 January 2020 through 31 July 2026. We distinguish source-evidence directness from validation maturity, which comprises discovery, analytical validation with independent replication, within-athlete longitudinal validation, and implementation testing against prespecified sport-relevant outcomes. We consider pathway-level interpretation of signals related to substrate use, recovery, and physiological stress, together with the metadata, repeated sampling, analytical quality control, and comparative testing required to establish value beyond established monitoring measures. Wearable biochemical sensing, continuous glucose monitoring, artificial intelligence, and multi-omics approaches may provide contextual information, but require analyte-specific calibration, transparent modeling, external validation, and demonstrated practical value. Metabolomics should therefore function as an explanatory layer in multimodal athlete monitoring while individual panels are evaluated in clearly defined sport and decision contexts.

## 1. Introduction

Metabolomics captures small-molecule responses to acute exercise and repeated training. In professional cyclists, metabolite profiles have been associated with endurance capacity [1]. Broader metabolomics and lipidomics studies show how substrate use, nutrition, recovery, and related exposures shape exercise physiology [2]. Athletic contexts such as heat, altitude, and travel can further modify these responses [3,4,5]. Exercise metabolomics primarily examines general physiological mechanisms. Sports metabolomics applies these approaches to athlete- and sport-specific questions, including training load, competition, recovery, and performance support [6,7,8]. The domains overlap, but a signal observed in general exercise studies does not by itself support athlete-level interpretation or decision-making. It must be validated for the intended sport and monitoring context.

The central translational question is whether exercise-responsive metabolites yield reproducible trajectories that improve monitoring of individual athletes. A useful signal must be analytically reliable, context-matched, longitudinally interpretable, and linked to a prespecified athlete-relevant outcome. These requirements cannot be inferred from an isolated pre–post or group comparison. Without additional evidence, such observations remain discovery-stage findings rather than monitoring tools. Athlete monitoring should therefore emphasize within-athlete patterns rather than isolated metabolites or universal thresholds.

Metabolic responses depend heavily on context. Lactate may reflect glycolytic flux, exercise intensity, clearance, or incomplete recovery. Beta-hydroxybutyrate may reflect prolonged exercise, fasting, carbohydrate restriction, or fat adaptation. Branched-chain amino acids, acylcarnitines, tricarboxylic-acid-cycle intermediates, inflammatory lipid mediators, creatine kinase, and myoglobin also require contextual interpretation [6,7,8]. Exercise modality, sampling time, training status, diet, sex, recent load, and environmental exposure can all alter their meaning. These signals can be physiologically informative, but they are not universal indicators of readiness or performance.

Previous reviews have addressed exercise and sports metabolomics, analytical standardization, non-invasive sampling, and multi-omics integration. A companion review by Lin et al., first published online on 27 July 2026 and captured within the updated three-database search window, examines analytical-platform selection, metabolite annotation and identification, quality control, pathway-level interpretation, and general biomarker-panel readiness across exercise and sports metabolomics [9]. It is cited solely to disclose the relationship between the reviews and clarify their respective scopes, rather than as primary evidence for athlete-monitoring claims.

The present review does not provide a comprehensive assessment of analytical platforms or metabolite annotation. Instead, it addresses the distinct translational question of whether an exercise-responsive metabolite or panel can support a defined athlete-monitoring decision. Its incremental contribution is to operationalize two complementary axes: the directness of source evidence, determined by relevance to the athlete population, sport, biological matrix, and outcome; and validation maturity, spanning discovery through implementation testing. It also specifies intended-use-dependent exit criteria for longitudinal athlete monitoring, including individual baseline characterization and within-athlete variation, and illustrates their application with representative cited examples. Metabolomic information should be interpreted alongside established measures of external load, heart rate, perceived exertion, sleep, nutrition, recovery status, and performance [10,11,12,13]. A section-level comparison with the companion review is provided in Supplementary Table S1.

The review follows a practical argument. It first distinguishes evidence directness from validation maturity, then considers monitoring-oriented platform selection, pathway interpretation, implementation constraints, and within-athlete longitudinal validation. It concludes with an evidence-readiness roadmap for context-specific application. This structure clarifies how mechanistic insight can be distinguished from monitoring utility.

## 2. Methodological Approach and Evidence-Readiness Framework

This structured narrative review used an explicit, non-systematic search and evidence-synthesis approach, described in the Supplementary Materials [14,15]. We searched PubMed, Scopus, and Web of Science for English-language, peer-reviewed articles published from 1 January 2020 to 31 July 2026. Search concepts combined exercise or sports metabolomics with athlete monitoring, recovery, fatigue, overreaching, training adaptation, substrate utilization, analytical platforms, metabolite annotation and identification, wearable biochemical sensing, artificial intelligence, and multi-omics integration. We also screened reference lists of key reviews and original studies. Earlier foundational reports were retained only when needed to support analytical standards, quality-control procedures, annotation frameworks, or athlete-monitoring principles.

Eligible records were English-language, peer-reviewed publications addressing metabolomics, metabonomics, lipidomics, or related small-molecule profiling in exercise or sports science. We considered original research, controlled human exercise studies, observational and interventional studies in sport-trained cohorts, longitudinal or repeated-measures studies, and analytical-method studies relevant to exercise. Reviews informed background, methodological context, and citation tracking, rather than serving as primary evidence for athlete-monitoring applications. Duplicate records were removed when identified. Studies in disease, aging, pharmacology, or preclinical models were retained only as indirect mechanistic context when they clarified exercise-responsive metabolic pathways.

The database-adapted search architecture and eligibility criteria are provided in the Supplementary Materials. The original workflow did not retain a prospectively maintained, database-level record log or a PRISMA flow diagram. Consequently, reliable counts of records identified, screened, and retained from the original searches cannot be reconstructed retrospectively. SPORTDiscus was not searched, which limits coverage of the sport-science literature. We therefore do not claim exhaustive or systematic retrieval. Supplementary Table S2 summarizes the extracted athlete-monitoring domains, and Supplementary Table S3 defines the four validation-maturity stages, their minimum evidence requirements, and their permitted athlete-monitoring conclusions.

Evidence was assessed on two independent axes. Source-evidence directness describes the relevance and transferability of source evidence to sport-specific application; it does not indicate validation maturity or application readiness. Level 1 evidence comprised human studies of sport-trained participants, defined here as trained recreational, competitive, or elite athletes, linked to training load, recovery, substrate use, fatigue, adaptation, or performance-related outcomes. This level identifies contextual directness, not a homogeneous population. Its transferability depends on training status, competitive level, sport discipline, sex, age, biological matrix, exposure, and outcome. Findings from recreationally trained cohorts should not be assumed to apply to elite athletes without supporting evidence. Level 1 evidence can support athlete-relevant interpretation and identify validation priorities, but does not itself establish a decision-ready marker. Level 2 evidence comprised controlled human exercise studies in healthy non-athletes; these studies inform general physiology but require validation before extrapolation to trained or elite athletes. Level 3 evidence included disease, aging, preclinical, technology-development, and other indirect mechanistic studies. We used Level 3 evidence to interpret pathways and generate hypotheses, not to support routine athlete monitoring or decision-making.

For clarity, this review treats fatigue, recovery, readiness, maladaptation, illness risk, and injury risk as related but distinct monitoring constructs. Fatigue denotes a context-dependent state of reduced capacity or increased perceived effort after a defined exercise exposure; it is not a metabolite-defined diagnosis. Recovery denotes the time-dependent restoration or reorganization of physiological and functional responses after a specified load. Readiness is an integrative, context-dependent judgment about suitability for a defined subsequent task and may be informed by, but is not equivalent to, recovery measures. Maladaptation denotes a persistent or adverse response to repeated training that requires longitudinal evidence relative to expected adaptation. Illness risk and injury risk are prospective incidence outcomes that require separately validated prediction studies with prespecified event definitions and time horizons. Metabolite panels may contribute to interpretation of these constructs only when they add value for the specified outcome; isolated signals do not establish fatigue, readiness, maladaptation, illness risk, or injury risk.

Established biomarker-development concepts offer useful precedents, but they do not constitute a regulatory standard for athlete monitoring. The FDA-NIH BEST Resource defines biomarker categories and emphasizes a specified context of use [16]. FDA drug-development-tool qualification asks whether a biomarker can be relied on for a defined interpretation and use in drug development [17]. Phased clinical biomarker models similarly distinguish discovery, assay evaluation, longitudinal assessment, and impact testing [18]. Our framework adapts these principles to athlete monitoring. Its sport-specific contribution is to assess source-evidence directness separately from validation maturity and to require context-matched longitudinal evidence and incremental value for a defined monitoring question. Athlete-centered evidence alone does not establish application readiness.

We assessed validation maturity independently of source-evidence directness. The four stages are discovery, analytical validation with independent replication, within-athlete longitudinal validation, and implementation testing. Table 1 translates these stages into intended-use-specific exit criteria for the matrix, platform, athlete population, and monitoring question. Progression to longitudinal validation requires documented analytical performance and independent replication. Progression to implementation testing requires repeated, context-matched athlete samples; characterization of analytical and within-athlete biological variation; and evidence of both reliability under prespecified stable conditions and responsiveness to a defined exposure. Application further requires external validation and added value beyond established monitoring measures. Individual baselines, reference intervals, and reference change values may inform interpretation only when their underlying data and assumptions are fit for purpose. Table 2 provides a worked application using sport-trained, controlled human exercise, and wearable-technology examples. Subsequent sections apply these criteria rather than repeating them.

## 3. Analytical Platforms for Sport-Science Questions

The companion review provides a comprehensive assessment of analytical platforms, metabolite annotation and identification, quality control, pathway-level interpretation, and general candidate-panel readiness [9]. This section therefore focuses on platform-selection considerations that directly affect serial athlete monitoring: the prespecified sport-science question, biological matrix, sampling frequency, pre-analytical control, analytical reproducibility, and identification or annotation requirements. In athlete-centered exercise and sports metabolomics, platform choice should follow these monitoring requirements rather than the number of detected features [10,12,13,25]. Figure 1 maps the monitoring objective and sport context to matrix selection, sampling design, pre-analytical control, platform choice, data processing, and pathway-level interpretation [26,27,28,29,30]. Table 3 summarizes the platform-selection considerations that most directly affect serial sampling and validation [27,28,29,30].

LC-MS is well suited to questions on substrate use, lipid and acylcarnitine remodeling, inflammation, recovery, and nutrition-related metabolic change. Its sensitivity and chemical breadth support targeted and untargeted analyses [27,28]. In athlete studies, LC-MS can profile amino acids, acylcarnitines, organic acids, ketone bodies, bile acids, lipid mediators, and complex lipids, providing pathway-level context for substrate selection, metabolic flexibility, inflammation, and recovery. For serial monitoring, interpretation requires transparent reporting of metabolite identification or annotation confidence, missing-data handling, internal standards, batch and matrix effects, normalization, and quality-control performance.

GC-MS is well suited to profiling volatile or derivatized metabolites in breath and urine, including signals related to substrate oxidation and oxidative stress. Established spectral libraries and reproducible workflows can facilitate repeated, minimally invasive sampling [31,32,33]. However, breath volatile organic compound profiles are influenced by ventilation, hydration, diet, ambient contamination, microbiome activity, sampling hardware, and data-processing procedures. In athlete monitoring, GC-MS is therefore best positioned as a complementary approach for physiological interpretation and method development, rather than a substitute for blood-based metabolic profiling.

NMR spectroscopy is well suited to longitudinal tracking of abundant metabolites associated with hydration, substrate use, and recovery. It has characterized plasma responses to acute resistance exercise in healthy older adults [34] and urinary metabolic responses to acute exercise [35]. High-throughput proton NMR enables reproducible, quantitative phenotyping of urine, serum, and plasma, although it is less sensitive than MS-based platforms for low-abundance metabolites [36,37]. This quantitative robustness is particularly valuable in repeated-measures designs, where within-athlete trajectories across training, tapering, competition, and recovery may be more informative than single between-group comparisons. Limited coverage of low-abundance metabolites and the need for specialist spectral interpretation remain important constraints.

IMS-MS adds gas-phase separation to improve discrimination among isobaric, co-eluting, and structurally related features. It can strengthen structural annotation and candidate-panel refinement when isomerism or co-elution limits identification confidence [19,23,38]. At present, however, IMS-MS is best regarded as a complementary technology rather than a routine platform for serial athlete monitoring. Its wider adoption in sport science will require expanded collision-cross-section databases, harmonized data-processing workflows, cross-platform comparability, and validation in sport-specific cohorts.

CE-MS efficiently separates highly polar and charged metabolites from small sample volumes. It can complement LC-MS and GC-MS in questions concerning energy metabolism, purine turnover, amino-acid utilization, and recovery-related change. Relevant analytes include amino acids, organic acids, nucleotides, and phosphorylated metabolites that can be incompletely resolved by other workflows. Routine use remains constrained by migration-time reproducibility, interface stability, sensitivity, lower lipid coverage, and limited interlaboratory standardization. It is best used as a complementary targeted or semi-targeted platform with rigorous sample preparation, migration-time control, internal standards, quality-control assessment, and matrix-specific validation [29,30].

## 4. Pathway-Level Interpretation for Training, Recovery, and Performance Support

In exercise and sports metabolomics, interpretation, rather than analytical sensitivity alone, is often the main translational challenge. Lists of altered metabolites rarely distinguish acute energy demand from adaptation, nutritional status, incomplete recovery, inflammatory stress, or technical variation. Athlete monitoring should therefore interpret metabolites as context-dependent components of coordinated pathways, rather than as isolated biomarkers.

Figure 2 integrates biological matrices, serial sampling, and contextual modifiers with pathway-level interpretation. It draws on evidence for endurance substrate selection [39], field-compatible sweat sampling [20], exercise-mode-specific plasma trajectories [40,41], and urinary metabolic responses to training and recovery [42]. The pathway modules include tricarboxylic-acid-cycle intermediates [21], lipid and ketone-body handling [43,44,45,46], amino-acid and protein-related processes [47,48,49], exercise-responsive kynurenine pathways [50,51,52,53], and complementary inflammatory and muscle-stress signals. Non-athlete inflammatory studies are used only as indirect mechanistic context [22,54], whereas exercise and football studies contextualize muscle-stress responses [55,56]. Within-athlete time courses and coordinated pathway patterns may reveal exercise-mode- and training-related responses that isolated metabolite measures miss [40,42,45].

Figure 2 is a conceptual, context-dependent pathway map, rather than a universal response chart. Exercise modality, intensity and duration, training status, dietary context, biological matrix, and sampling time can alter metabolite direction, magnitude, and recovery kinetics [39,40,42,44].

### 4.1. Pathway-Level Biomarker Readiness for Athlete Monitoring

A pathway-centered framework distinguishes mechanistic insight from monitoring utility. Table 4 differentiates analytical measurability from evidence readiness, because the availability of a robust assay does not itself establish that a metabolite feature is suitable for athlete monitoring. It organizes representative exercise-responsive metabolite classes and complementary physiological measures to support pathway-level interpretation. Lactate, glucose, pyruvate, β-hydroxybutyrate, acylcarnitines, and tricarboxylic-acid-cycle intermediates provide measurable signals of energy and substrate metabolism, but their interpretation remains context-dependent [21,39,40,42,43]. Amino-acid pathways require the same contextual approach [45,47,50]. Cortisol, IL-6, creatine kinase, and myoglobin provide complementary endocrine, inflammatory, and muscle-stress information rather than serving as standalone metabolomic biomarkers. Evidence from non-athlete inflammatory studies provides indirect mechanistic context only [22,54], whereas exercise and football studies help contextualize muscle-stress responses [55,56].

No single metabolite should be presumed to diagnose fatigue, overreaching, recovery readiness, or performance potential. Candidate metabolites and panels require biological plausibility, analytical reproducibility, and longitudinal validation. This includes demonstrating reliability under prespecified stable conditions, responsiveness to a defined exposure, and an association with a prespecified sport-relevant outcome. Their incremental value should be evaluated against established measures of recovery kinetics, substrate availability, training adaptation, fatigue-related function, and performance-related function. Interpretation must account for diet, sleep, circadian timing, infection, psychological stress, environmental exposure, and recent training load.

Exercise modality materially shapes pathway interpretation. Endurance exercise commonly influences carbohydrate and fatty-acid oxidation, tricarboxylic-acid-cycle activity, ketone metabolism, amino-acid turnover, and oxidative stress. In high-level endurance athletes, patterns involving tricarboxylic-acid-cycle intermediates, acylcarnitines, fatty-acid oxidation, and redox balance have been associated with endurance capacity and performance phenotypes [1,57]. These associations require sport-, sex-, and longitudinal validation before generalization. Resistance, sprint, and high-intensity interval exercise more prominently influence glycolytic metabolism, lactate and pyruvate dynamics, purine metabolism, amino-acid turnover, muscle-stress markers, and delayed repair. Interpretation should integrate exercise exposure with nutrition, recovery timing, inflammation, and muscle-stress context, rather than rely solely on acute muscle-damage markers [45].

Fatigue and recovery are multisystem states. Tryptophan–kynurenine metabolism may provide mechanistic context for exercise-related neuroimmune stress and fatigue-related processes [50,51,52,53]. IL-6, creatine kinase, and myoglobin are complementary inflammatory and muscle-stress measures. Evidence from overweight or sedentary adults may inform inflammatory mechanisms but is indirect for athlete monitoring [22,54], whereas exercise and football studies provide more contextually relevant evidence for muscle-stress responses [55,56]. Together, these measures may support interpretation of recovery or incomplete recovery when considered with exercise exposure, sampling time, and other monitoring data.

Recovery metabolomics should emphasize kinetics. A metabolite measured immediately after exercise may reflect acute metabolic demand, whereas the same metabolite measured 3, 24, or 48 h later may reflect clearance, repair, persistent stress, or delayed recovery. Serial sampling across defined recovery windows should be linked to functional outcomes, including power restoration, neuromuscular performance, soreness, perceived recovery, task-readiness ratings, and subsequent training quality [40,41,42]. Pyruvate, citrate, beta-hydroxybutyrate, and acetylcarnitine, together with acetyl-CoA where reliably quantified in the selected matrix, are most informative when interpreted alongside respiratory exchange, power output, heart rate, perceived exertion, dietary intake, training load, and recovery context [39,40,42,43,44].

Untargeted features and putatively annotated pathway signals should be regarded as discovery-stage findings until their identities and fit-for-purpose analytical performance have been established. Analytical validation requires appropriate chemical confirmation, such as MS/MS spectra, retention-time matching, or comparison with authentic standards, together with documented quantitative precision, quality-control performance, and, where appropriate, internal-standard use [27,28,58,59]. Longitudinal and implementation studies should then satisfy the intended-use-specific exit criteria outlined in Table 1.

The next question is whether a candidate metabolite panel provides interpretable information beyond established athlete-monitoring measures [10,12,25]. Relevant use cases include recovery following a defined load, assessment of substrate availability, and responses to repeated training or specific interventions. Nutrition-related questions, including carbohydrate periodization and supplementation, require sport-specific nutritional context [60], whereas low energy availability should be assessed within a REDs-informed clinical and sport framework [61]. When the intended use is within-athlete monitoring, interpretation requires longitudinal baselines because metabolic variation is shaped by both athlete-specific and exposure-related factors. Metabolomics has practical value only when longitudinal trajectories demonstrate explanatory, predictive, or decision-support value beyond established monitoring measures for prespecified outcomes. Otherwise, they should be regarded as mechanistic context rather than decision rules.

### 4.2. Mechanistic Evidence and Translational Boundaries

Exercise and sports metabolomics focuses on athlete monitoring, recovery assessment, training adaptation, substrate-use profiling, and interpretation for performance support. Evidence from adjacent fields, including aging biology, metabolic disease, immunometabolism, tissue-regeneration research, and exercise-mimetic studies, can nevertheless provide valuable mechanistic context for exercise-responsive pathways [62,63]. Such evidence may inform interpretation of mitochondrial biogenesis, inflammatory resolution, lipid handling, amino-acid metabolism, redox regulation, gut–host metabolism, and inter-organ crosstalk. In sport science, however, its role is explanatory and hypothesis-generating rather than a direct basis for athlete-management decisions.

Table 5 defines the boundary for translating these sources. Evidence from sport-trained cohorts is most direct for athlete monitoring, but transferability still depends on the reported training status, competitive level, sport discipline, sex, age, biological matrix, exposure, and outcome. Controlled exercise studies in healthy non-athletes support general physiological inference but require validation before extrapolation to trained or elite athletes. Disease, aging, preclinical, and technology-development studies are indirect evidence for routine athlete monitoring [62,63,64,65]. Source-evidence directness remains distinct from validation maturity.

This distinction is particularly important in multi-omics and exercise-mimetic research. Large-scale studies of human skeletal-muscle adaptation indicate that exercise responses vary by sex, exercise modality, tissue, and pathway. Circulating metabolites should therefore be interpreted in relation to tissue biology, training status, and exercise context [66]. Integrative metabolomics, multi-omics, and computational modeling can identify pathways associated with exercise adaptation [66,67,68], but most such findings remain mechanistic rather than directly applicable to athlete monitoring.

Accordingly, metabolites, pathways, and molecular signatures identified outside sport-trained cohorts should be regarded as mechanistic candidates until they have been validated in the intended athlete population. This validation requires standardized exercise protocols, appropriate sampling windows, and prespecified sport-relevant outcomes. Current evidence does not support the use of exercise mimetics or disease-derived metabolic targets to guide training, recovery management, field-based monitoring, or performance support. In this review, such sources inform pathway interpretation and study design, rather than direct athlete-monitoring or decision-support claims [62,63,64,65].

### 4.3. Wearable and AI-Assisted Monitoring: Constraints and Validation Needs

Wearable and near-real-time biochemical sensing may complement laboratory-based exercise and sports metabolomics. Continuous sweat-lactate monitoring has been proposed for sport applications [20], whereas wearable sweat-glucose sensing and on-body cortisol monitoring have been demonstrated mainly in proof-of-concept or health-monitoring settings [69,70]. Evidence readiness varies substantially across analytes, biological matrices, sensor designs, calibration procedures, and intended uses [71,72,73]. Interpretation of sweat-derived signals is influenced by sweat rate, skin contamination, hydration status, sampling site, sensor characteristics, and their relationship to blood or interstitial-fluid concentrations [20,70]. Heat and altitude introduce additional uncertainty in field settings [3,4]. These technologies may provide contextual information, but they do not replace laboratory-based metabolomics.

Continuous glucose monitoring (CGM) can characterize within-athlete glycemic patterns and exercise- or nutrition-related glucose responses in endurance athletes [74,75]. It may therefore help contextualize carbohydrate availability. However, CGM measures interstitial rather than blood glucose, and its interpretation must account for physiological lag, sensor stability, and the effects of movement and exercise intensity on measurement performance. In non-diabetic athletes, its practical value should be demonstrated through improved nutrition or training decisions, rather than inferred from the availability of continuous data alone [74,75]. At present, its most defensible role is to describe within-athlete patterns and generate nutrition-related hypotheses, rather than to define universal performance thresholds.

Sweat lactate requires similarly cautious interpretation. Local sweat-gland metabolism, sweat rate, sampling site, skin contamination, and sensor design may each affect the measured signal. Sweat lactate should therefore not be assumed to reflect blood-lactate kinetics. Until sport-specific studies relate sweat-lactate measures to blood lactate, power output, perceived exertion, and recovery outcomes across relevant environmental conditions, it should be regarded as a complementary field signal rather than a surrogate for established laboratory or field assessments [20].

AI and machine learning approaches can integrate metabolomic, wearable, physiological, nutritional, and performance data within longitudinal athlete-monitoring systems. Potential applications include detecting departures from an athlete’s baseline, classifying response patterns, prioritizing candidates for targeted validation, and supporting interpretation of high-dimensional datasets [76,77,78,79]. These applications do not, however, justify autonomous decision-making. In applied sport, models should be transparent, biologically plausible, independently evaluated, and interpretable to coaches, clinicians, and sport scientists. Reports should document sample size, feature stability, missing-data handling, confounding control, and internal and external validation performance [80,81]. Model-guided workflows should also demonstrate added value for prespecified sport-relevant outcomes.

Overfitting and limited transportability remain major risks. A model that performs well in a small or homogeneous cohort may not generalize across sports, training phases, nutritional states, or environmental conditions. This concern is especially relevant to sports metabolomics, where feature numbers are high, sample sizes are often modest, and athlete-level metadata may be incomplete [79,80,81]. AI-assisted metabolomics should therefore remain a tool for hypothesis generation and validation support, rather than autonomous decision-making.

In the near term, wearable and AI-assisted approaches may help flag deviations from an athlete’s own baseline, guide targeted laboratory testing, and inform discussions of nutrition, recovery, illness, hydration, environmental stress, and training load. Their value depends on integration with established measures, including heart rate, heart-rate variability, external load, perceived exertion, sleep, dietary intake, menstrual-cycle information where relevant, and performance testing [13,24,25]. Future studies should use harmonized sampling, laboratory calibration, analyte-specific validation, standardized metadata reporting, and outcome-based designs across athlete populations and exercise modalities. Wearable and AI-assisted metabolomics are developing components of integrated athlete monitoring, rather than isolated consumer metrics or standalone performance-prediction systems [13,26,72,73,82].

## 5. Implementation Barriers and Research Priorities for Applied Sport

Exercise and sports metabolomics lies between mechanistic discovery and applied athlete monitoring. Exercise-responsive metabolite signatures have been widely reported in human exercise studies [8,83]. The sport-trained evidence used to illustrate this review includes recreationally trained, competitive, and elite cohorts across sport disciplines, training phases, and biological matrices [45,84,85,86]. Table 2 applies the framework to seven representative cited examples; it is not a systematic inventory. All seven examples remain at discovery because at least one preceding Table 1 criterion is unmet. Small, heterogeneous cohorts [8,45], sparse repeated sampling, and limited sex- and sport-specific analyses constrain characterization of within-athlete trajectories [24,86,87]. Nutrition, energy availability, recovery, environmental exposures [3,4,5,12,60,61], and analytical reporting, quality control, normalization, and metabolite identification [27,28,58,88] should be incorporated into study design and reporting.

A common translational error is to equate biological informativeness with decision readiness. Metabolite changes may reflect substrate use and muscle-energy metabolism [39,40,41] or contribute to interpretation of inflammatory and muscle-stress responses when integrated with complementary measures [22,50,54,55,56]. This evidence base is heterogeneous, and findings from non-athlete populations provide only indirect context for athlete monitoring. Such findings may justify candidacy for recovery, nutritional-status, or maladaptation panels, but candidacy does not establish readiness for decision support. The next priority is to test candidate patterns against prespecified outcomes in the intended athlete population [10,12,13,25].

Studies intended to advance candidate features or panels should test validation, generalizability, and decision-relevant utility. They should prespecify the sport population, intended monitoring question, biological matrix, sampling schedule, nutritional and environmental context, quality-control procedures, annotation confidence, repeated-measures design, comparator monitoring measures, and validation outcomes [88,89]. Such designs move the field beyond descriptive profiling toward context-specific interpretation in athlete monitoring, recovery assessment, and performance-support research.

### 5.1. Sport-Specific Longitudinal Validation

Many studies rely on small cohorts, short follow-up periods, limited population diversity, and sampling schedules that do not span training cycles. Such designs cannot readily distinguish responses to the most recent exercise bout from baseline variability, nutritional effects, delayed recovery, environmental stress, and durable training adaptation. Larger, multicenter repeated-measures studies should quantify reliability under prespecified stable conditions, characterize analytical and within-athlete biological variation, and test responsiveness to defined training, recovery, nutritional, or environmental exposures. They should also assess transferability across sports, competitive levels, and training environments [45,86,87,89].

Longitudinal studies should sample athletes across baseline, intensified loading, recovery, tapering, competition, travel, and return-to-training phases. Such designs can characterize candidate patterns across these phases and test whether within-athlete trajectories are associated with prespecified sport-relevant outcomes, rather than only describe changes after performance has declined. Recent studies illustrate this need. A 48-week, six-time-point multi-omics study of 120 elite athletes linked changing energy balance to endocrine, metabolic, and microbiome responses [84]. Untargeted serum metabolomics also identified modality-specific baseline and exercise-response patterns across endurance athletes, sprinters, and bodybuilders [85]. These studies characterize candidate trajectories and contextual heterogeneity, but do not by themselves establish decision-ready monitoring panels.

Sport-specific interpretation is essential because the same metabolite pattern may have different meanings in endurance, sprint, resistance, team, combat, and mixed-modality sports. Future studies should integrate metabolomics with external and internal load, power or speed metrics, competition demands, diet, sleep, environmental exposure, menstrual-cycle information where relevant, and recovery measures. This design can characterize individualized metabolic trajectories and, where supported by appropriate data, assess context-specific reference intervals or decision criteria rather than assume generic metabolite thresholds.

### 5.2. Priority Gaps for Sport-Specific Translation

Several gaps limit biological interpretation, cross-study comparability, and practical translation in exercise and sports metabolomics [8,45,83,86]. Table 6 maps each priority gap to the validation-maturity stage or stages at which it should be addressed. Priorities include sport-specific longitudinal validation, rigorous control of nutrition and energy availability, and complete documentation of environmental and logistical stressors. They also include methodological harmonization, characterization of long-term adaptation, and validation of wearable, non-invasive, AI-assisted, and multi-omics approaches. These gaps identify requirements for improved interpretation and validation; they do not establish readiness for routine individualized athlete-management decisions.

Metabolite profiles are sensitive to biological context and pre-analytical conditions. Nutrition and energy availability can alter athlete physiology and the interpretation of metabolic data [60,61]. Menstrual-cycle phase, where relevant, may contribute to within-athlete variation [24]. Heat, altitude, and travel introduce further context-specific metabolic perturbations [3,4,5]. Recent exercise, hydration, sleep, supplement use, and competition demands should likewise be recorded as part of the athlete-monitoring context. Reproducibility also depends on standardized sampling and biofluid handling [90,91], analytical quality-control procedures [27,28], appropriate data normalization [88], and transparent metabolite-identification confidence [58]. These variables should be incorporated into the biological model and analytic plan, rather than treated as background detail.

Future studies should move beyond acute pre–post-exercise comparisons. Acute responses are informative but do not establish whether a metabolite signal reflects durable adaptation, incomplete recovery, or another context-specific response. A 2026 systematic review of high-intensity and sprint-interval exercise metabolomics identified substantial heterogeneity in protocols, matrices, and sampling designs [83]. A one-week endurance training-camp study likewise showed that metabolic and stress-related markers can change in different directions during the same exposure [92]. Longitudinal designs across training cycles should quantify reliability under prespecified stable conditions and responsiveness to defined exposures. They should also link trajectories to training load, recovery kinetics, performance, health status, and athlete-reported outcomes [45,86,87,93].

### 5.3. Interdisciplinary Integration and Outcome-Based Validation

Translation of exercise and sports metabolomics requires closer integration of analytical rigor with applied sport practice. Metabolomic signals should be interpreted alongside training design, recovery practices, nutritional strategies, environmental exposures, athlete-reported measures, and performance testing [3,4,5,60,61]. This athlete-centered approach is consistent with monitoring and consensus frameworks that call for joint interpretation of training load, recovery, fatigue, athlete-reported measures, and performance outcomes [10,11,12,13]. Menstrual-cycle information should likewise be considered where relevant [24].

Multi-omics integration may be informative when guided by sport-relevant biological questions rather than by data accumulation alone. Integrating metabolomics with lipidomics, proteomics, transcriptomics, epigenomics, microbiome profiling, wearable physiology, biomechanics, and nutrition records may help link biochemical signatures to substrate selection, mitochondrial remodeling, inflammatory stress, adaptation, and performance-related physiology [94]. Such work requires shared metadata templates, harmonized sample collection and processing, transparent normalization, batch-effect control, and clear reporting of pre-analytical factors [90,91,95].

Studies in elite and sport-trained cohorts illustrate this potential, but also show that responses vary according to sport phenotype, energy availability, sampling phase, and outcome definition [84,85]. Before integrated models can inform athlete-level decisions, they require interpretability, external validation, and biological plausibility.

Practical implementation ultimately requires evidence that metabolomics adds value beyond established monitoring tools. This outcome-centered approach should test whether metabolite panels improve interpretation or prediction of prespecified outcomes, including recovery, adaptation, illness or injury incidence, and performance, beyond training load, heart-rate variability, perceived exertion, sleep, and performance testing [10,11,12,13]. Added value must be demonstrated for prespecified sport-relevant outcomes rather than inferred from statistical separation alone.

## 6. Translating Exercise and Sports Metabolomics: An Evidence-Readiness Roadmap

Moving from discovery profiling to context-specific application requires staged validation. Table 5 defines the directness and transferability of source evidence, whereas Table 1 specifies cumulative, intended-use-specific exit criteria for validation maturity. Table 2 applies these two axes to representative cited examples. The five operational steps below guide study design and evaluation alongside the four validation-maturity stages outlined in Table S3. Direct relevance to sport does not, by itself, establish validation maturity or readiness for athlete monitoring.

Step 1 is to define the intended sport-science question before sample collection. The study design should prespecify the sport, exercise exposure, sampling matrix, sampling window, and decision context. Endurance-oriented studies should prioritize substrate selection, carbohydrate availability, fatty-acid oxidation, acylcarnitines, ketone bodies, tricarboxylic-acid-cycle intermediates, and delayed recovery [1,39,57]. Resistance, sprint, and high-intensity intermittent exercise require greater attention to glycolytic flux, phosphocreatine turnover, purine metabolism, amino-acid demand, muscle stress, inflammation, and repair kinetics [34,40,45,96]. In team sports, contact loading and repeated acceleration–deceleration may increase the relevance of muscle-stress markers, including myoglobin and creatine kinase [56]. Travel, sleep disruption, heat, altitude, and congested competition schedules should be modeled as explicit exposures rather than treated as generic background variables [3,4,5].

Step 2 is to align sampling design with biological timing. A single post-exercise sample rarely suffices for athlete monitoring. Sampling immediately or shortly after exercise can characterize acute, intensity-related responses, whereas serial sampling at baseline, after exercise, and across defined recovery windows can distinguish acute metabolic flux from delayed repair, persistent stress, or potentially maladaptive responses. For questions concerning training adaptation, repeated sampling across stable training, overload, tapering, competition, and return-to-training phases is more informative than isolated group comparisons [45,86,87,93].

Step 3 addresses the quantitative principles of within-athlete longitudinal validation by evaluating two distinct properties: the reliability of serial measurements under prespecified stable conditions and their responsiveness to a meaningful physiological change following a defined exposure. These properties are complementary but not interchangeable. Under stable conditions, analytical variation (CVa) should be estimated from fit-for-purpose assay and quality-control data, whereas within-athlete biological variation (CVi) should be estimated for the intended matrix, sampling conditions, and athlete population. Together with pre-analytical variation, these components define the expected variation in serial results when no relevant physiological change is anticipated. Separately, a candidate panel should be assessed for a reproducible, context-interpretable direction, magnitude, and time course of response to a prespecified training, recovery, nutritional, or environmental exposure, and for its association with a prespecified sport-relevant outcome.

A meaningful individual-level change therefore has two linked components. Statistical evidence of change asks whether a serial difference exceeds the expected analytical and biological variation; a reference change value (RCV) can support this assessment when its distributional assumptions, sampling conditions, and estimates of variation are appropriate. Physiological or monitoring significance further requires that the observed response be interpretable in its defined context, relate to a prespecified outcome, and add explanatory, predictive, or decision-support value beyond established monitoring measures. The index of individuality can help determine whether population-based reference intervals are informative relative to subject-based interpretation. Neither an RCV nor an individual reference interval alone establishes a physiologically meaningful, performance-relevant, or action-guiding change [97,98].

The number and timing of baseline samples required to characterize an individual cannot be specified universally. Baseline sampling is used to characterize measurement reliability and expected variation under stable conditions. It does not, by itself, establish sensitivity to a training, recovery, nutritional, or environmental exposure. The sampling plan should be justified according to the metabolite or panel, biological matrix, analytical precision, anticipated biological variability, training phase, and intended decision, with appropriate standardization for recent exercise, diet, hydration, time of day, environmental exposure, and other relevant sources of variation. Baseline observations should be sufficient to characterize an athlete’s context-specific distribution and support a prespecified comparison between stable-condition variability and exposure-related responses. Available athlete-specific biological-variation data remain limited and should not be generalized across metabolite panels, sports, or settings [97,98].

The Athlete Biological Passport (ABP) provides a relevant but bounded operational precedent for individualized longitudinal biochemical monitoring in sport. Its steroidal module follows serial urinary steroid profiles, including small-molecule steroid concentrations and ratios, using an adaptive model to derive individual reference limits from longitudinal data. Its hematological and endocrine modules similarly use standardized serial measurements, module-specific statistical interpretation, and expert review [99,100]. The ABP therefore illustrates how standardized sampling, documentation of confounders, and individualized longitudinal profiles can support athlete-level biochemical interpretation without reliance on universal population thresholds. However, its transfer to metabolomics is limited. The ABP is an anti-doping program with validated analytes, governed operating procedures, module-specific decision rules, and expert review. It does not validate targeted or untargeted metabolomic panels for training or recovery monitoring, nor does it provide performance, recovery, or training-decision thresholds. For sports metabolomics, the ABP is a design precedent for rigorous longitudinal validation and context-aware interpretation, not a direct application template or a substitute for outcome-based implementation testing.

Step 4 is to link metabolite panels to functional and contextual outcomes. Statistical separation between pre- and post-exercise samples alone does not establish practical utility. A candidate panel should provide added interpretive value for prespecified outcomes, such as power output, running speed, perceived exertion, neuromuscular function, recovery quality, illness symptoms, or training completion. It should also be evaluated against established monitoring measures, including training load, athlete-reported measures, and performance testing [10,11,12,13]. Panels without demonstrable incremental value should remain mechanistic research tools.

Step 5 is to test external validity and interpret decision thresholds cautiously. AI-assisted signatures may support hypothesis generation, but they require transparent reporting and independent evaluation before applied use [76,77,78,79]. Reports should document model-development and validation procedures [80,81]. Continuous sweat-lactate monitoring requires sport-specific validation [20], whereas sweat-glucose, cortisol, and other point-of-care platforms require analyte-, matrix-, and intended-use-specific validation [69,70,71,72,73]. Candidate panels and thresholds should be tested in cohorts that represent relevant variation in sex, sport, competitive level, nutritional state, and environmental exposure. Any reference interval or decision threshold must remain specific to the sport, sex, athlete population, and use context in which it has been validated [3,4,24,60,61].

Within integrated athlete monitoring, metabolomics is best positioned as an explanatory layer that contextualizes physiological change and prioritizes candidate panels for targeted evaluation. Application-ready use requires analytically robust, externally validated panels that add interpretable value for a defined decision context.

## 7. Conclusions

Exercise and sports metabolomics can strengthen athlete monitoring by explaining coordinated changes in substrate use, recovery, and physiological stress within a defined sport and monitoring context. At present, it is best used as an explanatory component of integrated monitoring rather than a source of universal biomarkers or standalone prescriptions. Translation depends on separating source-evidence directness from validation maturity and requires fit-for-purpose analytical performance, independent replication, repeated context-matched athlete sampling, and evidence of practical value for a defined monitoring question. Longitudinal interpretation must account for analytical and within-athlete biological variation, baseline design, and the assumptions underlying any RCV or individual reference interval. Progress will depend on prespecified use cases, complete contextual metadata, comparison with established monitoring measures, and outcome-focused testing in the intended athlete population; current reports remain valuable for hypothesis generation and mechanistic interpretation but do not, individually or collectively, establish application-ready athlete-monitoring panels [101,102,103,104,105,106].

## Figures and Tables

**Figure 1:**
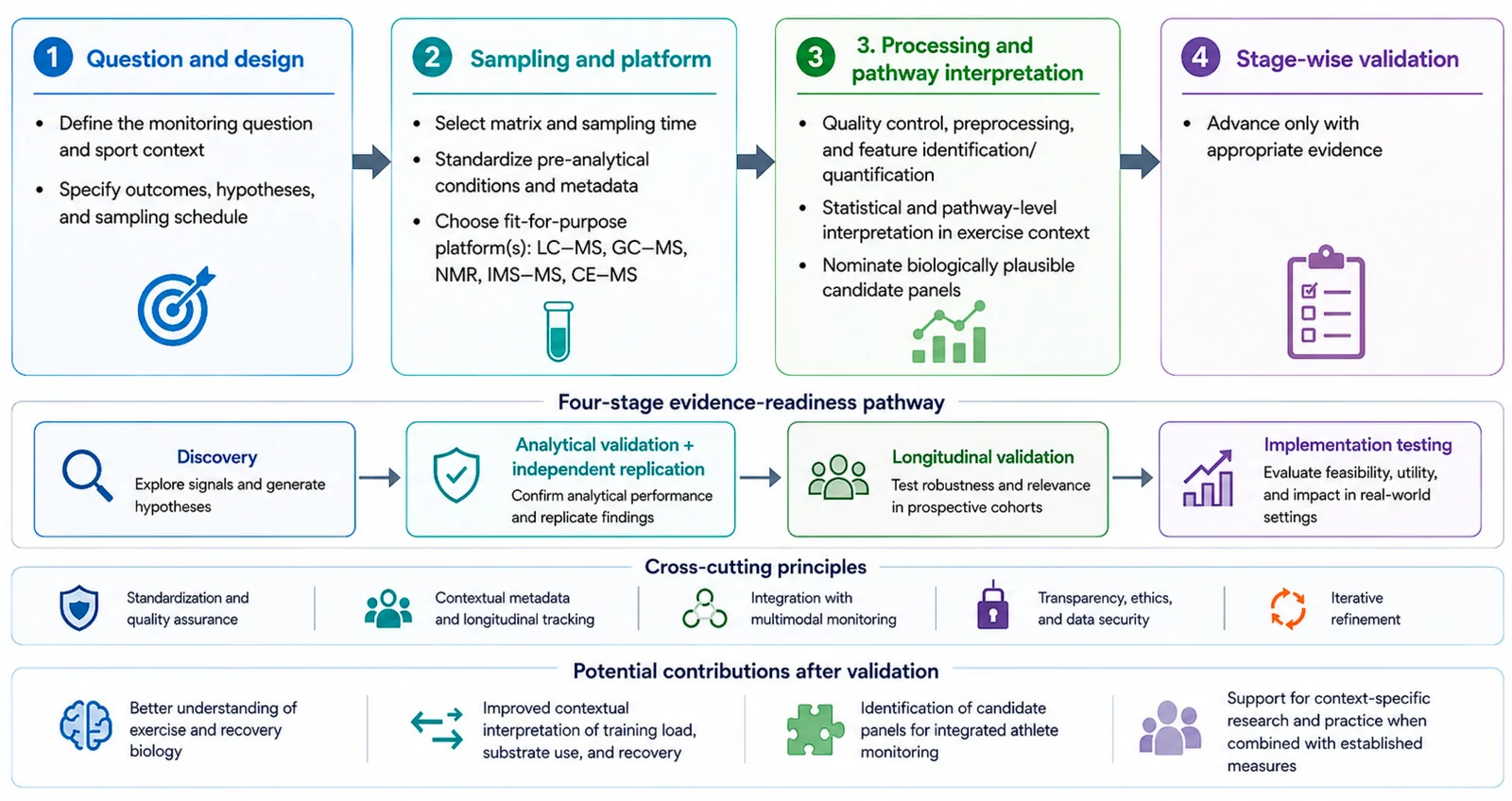
Application-oriented workflow for athlete-centered exercise and sports metabolomics. A defined sport-science question and athlete context guide biological-matrix selection, sampling design, pre-analytical control, platform choice, data processing, and pathway-level interpretation. Candidate panels may make context-specific monitoring contributions only after validation. Standardization, quality assurance, within-athlete longitudinal tracking, multimodal integration, data transparency, ethics, and data security are cross-cutting requirements.

**Figure 2:**
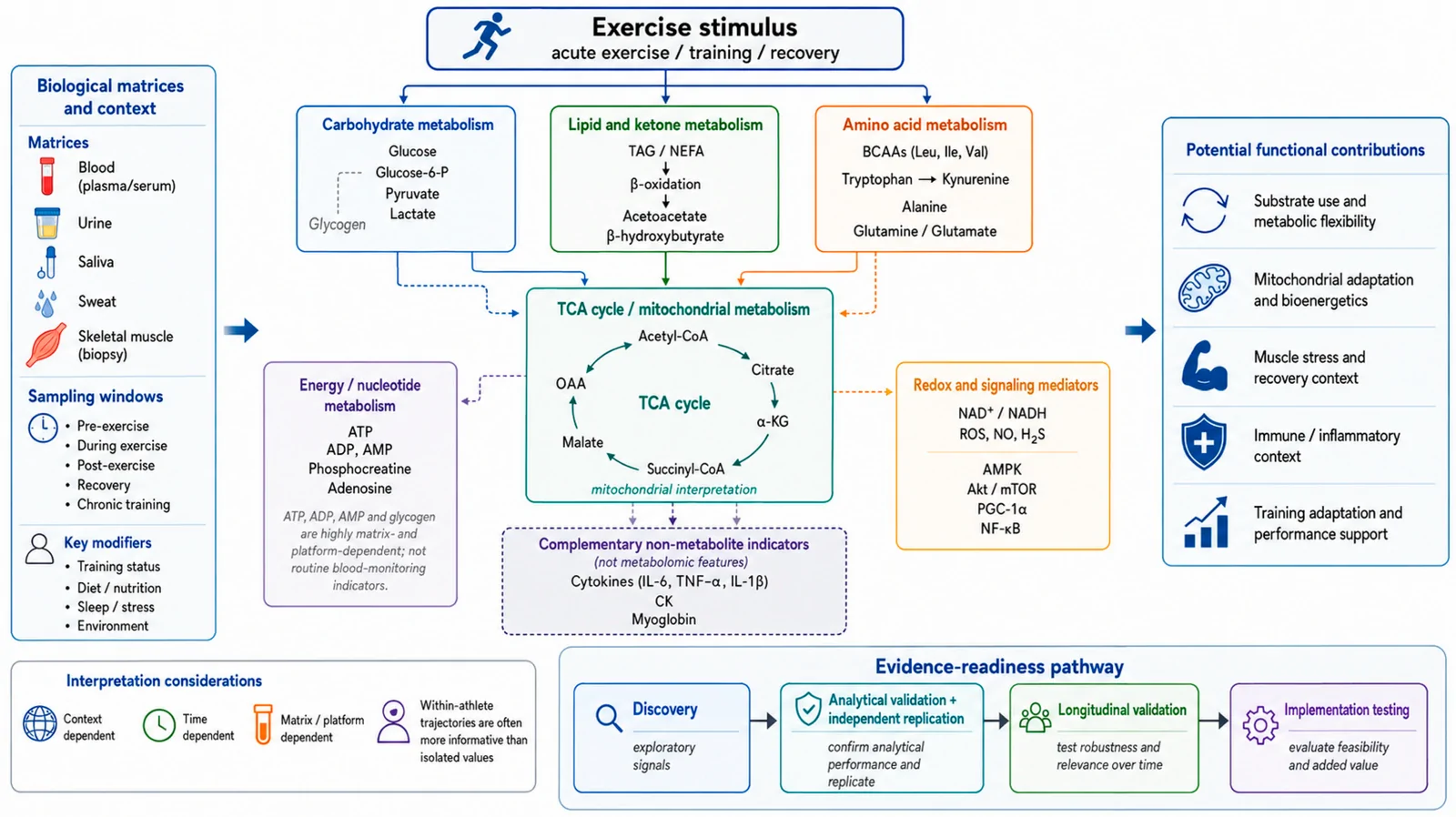
Context-dependent pathway map for athlete-centered exercise and sports metabolomics. The schematic links exercise exposure, contextual modifiers, biological matrices, and serial sampling to pathway-level interpretation of candidate metabolite patterns. It illustrates how pathway-level evidence can be considered from discovery through implementation testing; it does not imply that individual metabolites are application-ready monitoring tools.

**Table 1:** Four-stage validation-maturity framework for exercise and sports metabolomics.

Stage	Purpose and Minimum Evidence	Operational Exit Criterion for Progression	Expected Output and Readiness Boundary
1. Discovery	Identify a biologically plausible feature or candidate panel for a prespecified sport-science question; document matrix, sampling window, preprocessing, missingness, batch effects, pooled quality-control performance, and annotation confidence.	Before analytical validation, define the intended use and lock the candidate feature or panel. Establish sufficient chemical identity or annotation confidence to select a fit-for-purpose assay and a prespecified quality-control plan for the intended matrix.	Hypothesis-generating feature or pathway signal; not a monitoring marker or decision rule.
2. Analytical validation with independent replication	Confirm chemical identity as appropriate; evaluate assay precision, bias or accuracy, linearity or reportable range, sensitivity, matrix effects, carryover where relevant, internal standards, and quality-control performance for the intended use.	The prespecified assay and quality-control criteria are met, and the locked feature or panel is replicated in an independent, sport-relevant cohort using transparent feature selection and model procedures.	Replicated candidate feature or panel requiring within-athlete longitudinal validation.
3. Within-athlete longitudinal validation	Use repeated, context-matched athlete samples across defined baseline, exercise, recovery, and training-phase windows. Under conditions prespecified as physiologically stable, characterize analytical variation (CVa) and within-athlete biological variation (CVi) to establish reliability. Separately assess sensitivity to a prespecified physiological exposure by evaluating the reproducibility, direction, magnitude, and time course of response in relation to a prespecified sport-relevant outcome. The number and timing of baseline samples must be justified for the intended analyte, matrix, athlete population, and decision context.	Before implementation testing, show both (i) reliability under prespecified stable conditions and (ii) a reproducible, context-interpretable response to the predefined exposure that is linked to a prespecified sport-relevant outcome. Assess serial change against relevant CVa/CVi, for example through an RCV or individualized-reference approach when its assumptions are met, and compare its explanatory or predictive value with established monitoring measures. An RCV or individual reference interval indicates change relative to expected variation; neither is, by itself, a performance or training threshold.	Longitudinally supported candidate panel requiring implementation testing.
4. Implementation testing	Test practical sampling, turnaround, quality assurance, interpretability, calibration, and generalizability in independent athlete populations representative of the intended setting.	The panel provides feasible and interpretable incremental value beyond established monitoring measures for a predefined decision, without being used in isolation. Any reference interval or threshold remains specific to its intended context.	Context-specific application-ready panel only after added value is demonstrated; not a universal threshold or standalone decision.

Note: This table classifies validation maturity. Each exit criterion must be prespecified for the intended matrix, analytical platform, athlete population, and decision context; the criteria are not universal numerical cut-offs. Implementation testing requires evidence that a panel adds value beyond established monitoring measures for a defined, context-specific decision.

**Table 2:** Worked application of source-evidence directness and validation maturity to representative evidence examples.

Candidate and Intended Question	Cited Evidence and Source-Evidence Directness	Current Validation-Maturity Stage and Justification	Principal Gap and Next Required Exit Criterion
Whole-blood lactate-, TCA-cycle-, and CoA-precursor response pattern for interpreting acute workload and endurance capacity in professional cycling.	Targeted and untargeted whole-blood metabolomic responses before and after a graded test in 21 male WorldTour cyclists were linked to power-adjusted lactate accumulation. Level 1: elite athletes, sport-specific exposure, and performance-linked physiology [1].	Discovery. The study identifies associated metabolites in one team cohort but does not report a locked panel replicated in an independent athlete cohort.	No independent analytical replication or longitudinal outcome validation. Next: confirm a prespecified targeted panel and replicate it in an independent cyclist cohort before serial athlete testing.
Plasma metabolite pattern after resistance versus endurance exercise for interpreting mode-specific acute metabolic responses.	Randomized, within-subject crossover plasma metabolomics in humans under controlled exercise. Level 2: controlled human exercise evidence without a defined athlete-monitoring population or applied athlete outcome [19].	Discovery. The time-resolved signatures clarify general exercise physiology but have not been replicated as a monitoring panel in trained athletes.	Athlete-specific transferability and outcome linkage are untested. Next: replicate a locked candidate panel in a sport-relevant cohort using matched exercise and sampling conditions.
Urinary NMR metabolite pattern after HIIT and whole-body cryostimulation for interpreting acute recovery-related responses.	Urine NMR study in recreational athletes exposed to exercise, HIIT, and cryostimulation. Level 1: athlete-centered human evidence, but with limited transferability to trained competitive populations and no decision outcome [20].	Discovery. The report identifies acute urinary responses but does not provide independent replication, a locked targeted panel, or longitudinal outcome testing.	Population and context are narrow, and recovery utility is untested. Next: analytical replication followed by repeated measurements across defined training and recovery windows.
Lactate, acetylcarnitine, fatty-acid, xanthine, and glutamate response pattern for comparing standardized exercise responses in endurance- and strength-trained athletes.	Pilot plasma-metabolomics study of endurance- and strength-trained athletes versus sedentary participants after standardized exercise. Level 1: trained-athlete evidence with exercise-specific sampling, although the study is exploratory and not tied to a defined monitoring decision [21].	Discovery. Athlete groups are included, but the reported metabolites have not been analytically replicated as a predefined panel or tested longitudinally.	Small exploratory evidence base and no outcome-linked serial validation. Next: independent replication of a prespecified panel, then within-athlete testing across training phases.
Dried-blood-spot exertion profile, including lactate, carboxylic acids, fatty acids, and acylcarnitines, for contextualizing laboratory and race demands in elite cycling.	Metabolomics in 28 World Tour male cyclists during laboratory tests and in five cyclists during a seven-stage race. Level 1: elite athlete evidence with sport-specific field context and performance-related observations [22].	Discovery. Field feasibility and biological relevance are shown, but the signature was not evaluated as a locked, independently replicated monitoring panel with added value over standard measures.	No external cohort, decision-comparator analysis, or implementation outcome. Next: replicate the prespecified panel across independent teams and test incremental value beyond workload, lactate, and performance measures.
Wearable sweat-lactate signal for contextual field monitoring of exercise intensity and recovery.	Narrative review of continuous sweat-lactate monitoring in sport. Level 3: technology-focused evidence; it does not provide direct validation of a defined athlete outcome or decision application [23].	Discovery. The signal is technically plausible, but sweat rate, sampling site, contamination, and sensor characteristics limit inference from sweat lactate to blood or performance measures.	Analytical and biological correspondence remain insufficiently established. Next: validate the sensor against reference methods and sport-relevant outcomes under defined environmental and hydration conditions.
Multi-omic energy-balance response profile, including metabolomic pathways, for interpreting training-related energy availability in elite athletes.	Six time points across a 48-week training cycle in 120 elite athletes stratified by sport type. Level 1: athlete-centered longitudinal evidence with training-related and energy-balance outcomes [24].	Discovery. The longitudinal design directly informs candidate trajectories, but the report does not establish an independently replicated, analytically validated metabolite panel for a defined monitoring decision.	Athlete-centered longitudinal evidence does not replace preceding analytical replication or demonstrate incremental decision value. Next: lock and replicate a targeted panel, then evaluate added value against established nutritional and training-load measures.

Note: This table classifies the source context and current maturity of the cited examples. All remain at the discovery stage.

**Table 3:** Monitoring-oriented platform-selection considerations for serial athlete-metabolomics studies.

Platform	Best-Suited Sport-Science Questions	Common Matrices	Main Strengths	Monitoring-Specific Role and Serial-Sampling or Validation Constraint
LC-MS	Substrate utilization; lipid and acylcarnitine remodeling; inflammatory responses; recovery panels; nutrition-related metabolic changes	Plasma, serum, blood, urine, saliva, sweat, tissue extracts	High sensitivity; broad metabolite and lipid coverage; suitable for targeted and untargeted workflows; useful for pathway discovery and panel validation	Broad discovery, lipidomics, acute-response and recovery-kinetic studies, and targeted validation. Report internal standards, pooled quality controls, batch and matrix effects, missingness, and annotation confidence.
GC-MS	Breath or urine profiling; volatile metabolites; substrate oxidation; oxidative-stress-related signals; minimally invasive repeated sampling	Breath, urine, blood, tissue extracts	Mature spectral libraries; good reproducibility for volatile or derivatized compounds; useful for breath and urine-based monitoring studies	Breath VOCs, volatile or derivatized metabolites, organic acids, and fatty acids. Control ventilation, diet, hydration, ambient contamination, and derivatization variability.
NMR spectroscopy	Longitudinal tracking of abundant metabolites; hydration status; substrate use; recovery trajectories; within-athlete repeated-measures designs	Plasma, serum, urine, saliva, sweat	Quantitative reproducibility; minimal sample preparation; non-destructive analysis; well suited to serial sampling and within-athlete comparison	Serial within-athlete profiling of abundant metabolites. Lower sensitivity and coverage than MS, but strong quantitative reproducibility with standardized acquisition and processing.
IMS-MS	Structural discrimination of complex or isobaric features; lipid and acylcarnitine profiling; improved annotation confidence	Plasma, serum, urine, sweat, blood	Additional separation dimension; improved feature discrimination; collision cross-section information; useful for structurally similar metabolites	Emerging complementary dimension for structural discrimination and annotation refinement. Requires collision-cross-section resources, harmonized processing, and sport-specific validation.
CE-MS	Polar and charged metabolite profiling; amino-acid use, nucleotide and purine turnover, energy metabolism, and recovery-related pathway changes	Plasma, urine, sweat, tissue extracts	High-efficiency separation of polar and ionic metabolites from small volumes; complements LC-MS and GC-MS	Targeted or semi-targeted validation of polar metabolites. Requires migration-time control, interface stability, quality-control assessment, internal standards, and matrix-specific validation; lower lipid coverage and limited interlaboratory standardization.

Note: This table is intentionally limited to platform-selection issues that influence serial athlete monitoring and does not reproduce the comprehensive analytical-platform, annotation, and identification discussion [9]. Platforms are complementary rather than interchangeable. Selection should match the sport-science question, sample matrix, target metabolite class, expected concentration range, sampling frequency, annotation or identification requirements, quantitative objective, and validation stage.

**Table 4:** Representative exercise-responsive metabolites and complementary physiological markers: pathway-level interpretation and validation boundaries.

Category or Marker Type	Representative Metabolite or Complementary Marker	Main Pathway or Physiological Context	Exercise-Related Interpretation	Evidence-Readiness Boundary and Validation Needs
Glycolytic and carbohydrate-related metabolites	Lactate, glucose, pyruvate	Glycolytic flux, carbohydrate availability, redox-linked energy metabolism	Useful for acute carbohydrate-flux interpretation when workload and sampling time are standardized	Measurable by targeted or untargeted workflows. Monitoring use requires within-athlete longitudinal validation and added value for sport-relevant outcomes; individual signals remain context-dependent [20,39,40,41].
Ketone, lipid, and mitochondrial substrate-handling metabolites	Beta-hydroxybutyrate, acylcarnitines, acetylcarnitine, citrate, selected TCA-cycle intermediates	Substrate switching, fatty-acid oxidation, mitochondrial substrate handling, anaplerosis	Especially informative in prolonged exercise and recovery, but response direction is context-dependent	Discovery-to-candidate-panel features. Acetyl-CoA and labile intermediates are difficult to quantify in routine biofluids; use matrix-specific targeted, longitudinal, and outcome-linked validation [21,42,43,44,46].
Amino-acid and nitrogen-metabolism metabolites	Amino acids, BCAAs, Tryptophan–kynurenine metabolites	Amino-acid oxidation, nitrogen metabolism, dietary intake, muscle repair, neuroimmune signaling	Can inform pathway-level interpretation but should not be treated as universal fatigue or recovery thresholds	Discovery-to-candidate-panel features. Dietary context, repeated measures, chemical-confidence reporting, and outcome-linked validation are required; these are not universal thresholds [47,50,51,52,53].
Energy/nucleotide and redox/signaling metabolites	Purine-related metabolites (hypoxanthine, inosine, xanthine, urate); glutathione- and nicotinamide-related features	ATP turnover and purine salvage; redox buffering and redox-sensitive signaling	Can support interpretation of high-intensity energy turnover and recovery, but response direction and recovery kinetics are matrix- and context-dependent	Discovery-to-candidate-panel features. Exercise-mode-, matrix-, and training-status-dependent findings require targeted confirmation, within-athlete longitudinal replication, and links to sport-relevant outcomes before application-ready use [40,42,45].
Complementary physiological markers (not metabolites)	Cortisol, IL-6, creatine kinase, myoglobin	Endocrine strain, inflammation, muscle stress and repair	Provide context for metabolomic interpretation rather than standalone metabolomic biomarkers	Tryptophan–kynurenine pathways provide mechanistic context [50,51,52,53]. Muscle-stress variables are complementary measures [55,56]. Inflammatory evidence from non-athlete cohorts is indirect and cannot establish athlete-monitoring utility [22,54]. Longitudinal, outcome-linked validation is required; no standalone readiness decision.

Note: Metabolites should be interpreted as pathway-level, context-dependent signals rather than isolated indicators of performance or readiness. Their meaning depends on exercise modality, intensity, duration, training status, nutritional state, sample matrix, sampling time, sex, environment, and recent load history. Evidence readiness must be assessed for a defined feature, panel, or monitoring application in its intended context; it cannot be inferred from an individual analyte in isolation.

**Table 5:** Source-evidence directness framework for interpreting exercise and sports metabolomics studies.

Source-Evidence Directness	Assignment Criteria and Examples	Appropriate Inference	Key Limitations and Validation Needs
Level 1: athlete-centered human evidence	Human studies in sport-trained participants, reported as trained recreational, competitive, or elite; sport-relevant exercise exposures; defined sampling windows; links to load, recovery, adaptation, or performance outcomes	Supports athlete-relevant interpretation and validation prioritization; not by itself evidence for implementation readiness	Training status, competitive level, sport discipline, sex, age, matrix, exposure, and outcome define transferability; do not extrapolate recreational findings to elite athletes without supporting evidence
Level 2: controlled human exercise evidence	Healthy non-athlete participants under controlled exercise protocols	Supports general physiological and pathway inference	Requires validation before extension to trained or elite athletes; ecological and longitudinal relevance may be limited.
Level 3: indirect mechanistic evidence	Disease, aging, preclinical, tissue-specific, technology-development, or other adjacent contexts	Supports mechanism and hypothesis generation	Not direct evidence for athlete monitoring; requires athlete-centered validation with standardized protocols, sampling windows, and functional outcomes

Note: Source-evidence directness is independent of validation maturity. Level 1 evidence can support athlete-relevant interpretation and validation prioritization, but does not establish an application-ready monitoring marker.

**Table 6:** Priority research gaps, recommended actions, and primary evidence-readiness stages for sport-specific metabolomics.

Priority Gap	Why It Matters for Sport Application	Recommended Research or Reporting Action	Primary Validation- Maturity Stage(s)
Limited sport-specific longitudinal validation	Many candidate markers have not been tested across training phases, sport disciplines, competitive levels, or real-world recovery and performance outcomes	Use repeated-measures designs across baseline, intensified loading, recovery, tapering, competition, travel, and return-to-training phases; characterize within-athlete baselines and context-specific trajectories for subsequent validation	3. Within-athlete longitudinal validation
Inadequate nutrition and energy-availability control	Carbohydrate availability, low energy availability, ketogenic or low-carbohydrate diets, supplements, and pre-sampling meals can dominate metabolite profiles	Record and standardize pre-sampling nutrition, hydration, supplement use, carbohydrate availability, and energy status; integrate metabolomics with dietary records and substrate-use measures	1. Discovery. 3. Within-athlete longitudinal validation
Understudied environmental and logistical stressors	Heat, altitude, humidity, travel, jet lag, sleep disruption, and competition stress can alter substrate use, inflammation, hydration, oxidative stress, and recovery kinetics	Document environmental conditions, travel, sleep, time of day, competition context, and recovery state; conduct ecologically valid studies in real training and competition settings	1. Discovery. 3. Within-athlete longitudinal validation
Insufficient methodological harmonization	Differences in sampling, biofluid handling, extraction, normalization, batch correction, and annotation limit reproducibility and cross-study comparison	Report sample timing, fasting status, recent exercise, menstrual-cycle phase where relevant, sample handling, analytical workflow, quality-control performance, annotation confidence, missing data, and normalization procedures	1. Discovery. 2. Analytical validation with independent replication
Limited evidence on long-term adaptation	Acute responses are better characterized than chronic changes in mitochondrial remodeling, lipid utilization, substrate flexibility, and sustained metabolic plasticity	Track metabolite trajectories across training cycles and link them to training load, recovery kinetics, performance testing, health status, and athlete-reported outcomes	3. Within-athlete longitudinal validation
Uneven validation of wearable, non-invasive, and AI-assisted tools	Field-compatible tools are promising but may be affected by sensor drift, matrix differences, overfitting, and limited external validation	Calibrate wearable and non-invasive measures against laboratory methods; externally validate AI models; test whether these tools add value beyond established monitoring variables	2. Analytical validation with independent replication. 4. Implementation testing
Limited multi-omics and outcome integration	Metabolomics alone may not fully explain adaptation, fatigue, recovery, or maladaptation	Integrate metabolomics with lipidomics, proteomics, transcriptomics, microbiome data, wearable physiology, biomechanics, nutrition records, and performance outcomes, while maintaining interpretability and external validation	1. Discovery. 3. Within-athlete longitudinal validation. 4. Implementation testing

Note: The listed validation-maturity stage(s) indicate where each gap should first be addressed or formally tested, rather than discrete tasks that occur only once. Discovery-stage gaps primarily concern reporting, study design, and hypothesis generation; later-stage gaps should be addressed by meeting the corresponding stage-specific exit criteria in Table 1. Contextual factors, including nutrition and environmental exposure, require attention throughout the evidence-development pathway.

## Data Availability

No new data were created or analyzed in this study. Data sharing is not applicable to this article.

## Supplementary Materials

The following supporting information can be downloaded at: https://www.mdpi.com/article/10.3390/metabo16100704/s1, Table S1: Scope and section-level distinction between the published companion review and the present narrative review; Table S2: Minimum extraction domains for athlete-monitoring interpretation; Table S3: Four validation-maturity stages and permitted athlete-monitoring conclusions.
