# Supplementary material for: Metabolomics for Athlete Monitoring: An Evidence-Readiness Framework for Contextual Validation
Source: Metabolites. 2026 Sep 23;16(10):704. doi: 10.3390/metabo16100704 (PMC13706814; doi:10.3390/metabo16100704)
Supplement: Supplementary file 1 [file metabolites-16-00704-s001.zip › metabolites-4542551-supplementary.pdf]

Supplementary Material

Structured Search Strategy and Evidence-Readiness Framework for

"Metabolomics for Athlete Monitoring: An Evidence-Readiness Framework for Contextual Validation"

S1. Purpose and scope of the supplementary methodology

This supplementary material sets out the structured search, screening, and evidence-synthesis approach used in this narrative review of exercise and sports metabolomics for athlete monitoring. It explains why many exercise-responsive features remain mechanistic or discovery-stage observations, and what evidence is required before a metabolite panel can inform athlete-centered interpretation. The review was structured but narrative in design. It was not a systematic review and did not include a PRISMA flow diagram, formal risk-of-bias assessment, or quantitative meta-analysis.

The methodology followed transparent narrative-review principles, with explicit search sources, eligibility criteria, synthesis logic, and a prespecified framework for athlete-monitoring evidence. Given the sensitivity of metabolomics to pre-analytical handling, analytical reproducibility, annotation confidence, and quality-control procedures, we assessed two independent dimensions: source-evidence directness, reflecting relevance to athletes, and validation maturity, reflecting analytical quality, replication, longitudinal stability, and implementation utility.

S2. Literature search strategy and screening approach

The original core searches of PubMed, Scopus, and Web of Science covered English-language, peer-reviewed journal articles published from 1 January 2020 to 31 July 2026. PubMed covered biomedical, metabolic, and exercise-physiology literature. Scopus extended coverage to analytical chemistry, sport science, and interdisciplinary research, while Web of Science supported citation-based searches. SPORTDiscus was not searched; this limits coverage of sport-science literature and is acknowledged as a methodological limitation. Earlier methodological papers, consensus statements, and background references were retained only when needed to support established concepts, reporting standards, analytical procedures, or athlete-monitoring frameworks. The companion review cited as Reference 9 in the main text was first published online on 27 July 2026 and was within the updated three-database search window. It is cited solely to disclose the relationship and compare scope, not as primary evidence for athlete-monitoring claims; Supplementary Table S1 provides this delineation.

The original search used three concept blocks joined by Boolean operators: exercise or sport context, metabolomics-related profiling, and athlete-monitoring or outcome terms. The core search architecture was: (exercise OR training OR athlete OR sport) AND (metabolomics OR metabonomics OR lipidomics OR "small-molecule profiling") AND (recovery OR fatigue OR overreaching OR adaptation OR performance OR monitoring). Topic-specific searches added terms for analytical platforms (LC-MS, GC-MS, NMR, IMS-MS, CE-MS), biological matrices (blood, urine, saliva, sweat, breath), and applications such as wearable biochemical sensing, continuous glucose monitoring, artificial intelligence, and multi-omics. Field tags, controlled vocabulary, and syntax were

adapted for each database, while the conceptual structure was retained. The June-July 2026 update applied the same three-block architecture and publication-date filter across PubMed, Scopus, and Web of Science; it was not a new systematic search or a reconstruction of the original record-level screening process.

We also screened the reference lists of relevant reviews and original studies. Priority was given to sport-trained cohorts with reported training status and competitive level, repeated or longitudinal sampling, clearly defined exercise protocols, specified analytical platforms, and outcomes related to recovery, fatigue, adaptation, substrate use, performance, or physiological monitoring. Controlled human exercise studies informed pathway interpretation. Studies of disease, aging, preclinical models, or technology development were used only as indirect mechanistic context.

Table S1. Scope and section-level distinction between the published companion review and the present narrative review

Aspect	Companion review	Present narrative review
Primary purpose	Analytical and interpretative overview of exercise and sports metabolomics.	Translation of metabolomic findings to defined athlete-monitoring questions.
Core coverage	Platforms, annotation and identification, quality control, pathway-level interpretation, and general panel-readiness prerequisites.	Source-evidence directness, validation maturity, within-athlete longitudinal validation, and context-specific implementation.
Longitudinal focus	Repeated-measures and validation requirements considered as broad readiness principles.	Operational exit criteria, within-athlete baselines and variation, and fitness of RCV or individual-reference-interval approaches for intended use.
Applied endpoint	Biological interpretation and candidate-panel development.	Added value beyond established monitoring measures and feasibility for a specified monitoring decision.
Relationship to this synthesis	First published online on 27 July 2026; within the updated PubMed, Scopus, and Web of Science search window through 31 July 2026.	Cited only for transparent disclosure and scope comparison; not used as primary evidence for athlete-monitoring claims.
Section-level emphasis	Broad analytical and interpretative coverage of platforms, annotation and identification, quality control, pathways, and general panel-readiness prerequisites.	Section 2: source-evidence directness, validation maturity, and exit criteria; Section 3 and Table 3: monitoring-oriented platform selection and serial-sampling constraints; Section 4: pathway interpretation and candidate-panel readiness; Sections 5-6: implementation barriers, within-athlete longitudinal validation, and a context-specific translation roadmap.

Note: Source-evidence directness describes the relevance and transferability of the study context, whereas validation maturity describes the stage of evidence development and the permitted athlete-monitoring inference. Level 1 comprises human evidence from sport-trained participants, reported as trained recreational, competitive, or elite; its transferability depends on the recorded cohort characteristics and does not imply that findings in recreational participants apply to elite athletes. Level 1 can support athlete-relevant interpretation and identify validation priorities. It does not, on its own, establish analytical validation, longitudinal validity, implementation utility, universal thresholds, or athlete-management decisions made in isolation.

S3. Eligibility Criteria

Eligible studies were peer-reviewed, published in English, and examined metabolomics, metabonomics, lipidomics, or related small-molecule profiling in exercise or sports science. We included original research, controlled human exercise studies, observational and interventional studies in sport-trained cohorts, longitudinal or repeated-measures studies, and analytical-method studies relevant to exercise. Reviews were used for background, methodological context, and citation tracking, not as primary evidence for athlete-monitoring applications.

Records were screened against the review aims and eligibility criteria, with duplicate records removed where identified. We retained studies that informed athlete monitoring, recovery biology, exercise metabolism, analytical methodology, or translational interpretation. The original workflow did not include a prospectively maintained database-level record log; consequently, reliable numbers of records identified, screened, and retained from the original searches cannot be reconstructed retrospectively. The June-July 2026 update does not permit retrospective reconstruction of those counts. The process supported a structured narrative synthesis rather than exhaustive systematic retrieval, and we did not include a PRISMA flow diagram.

We excluded non-peer-reviewed studies; conference abstracts lacking sufficient methodological detail; opinion pieces without substantive evidence synthesis; studies unrelated to exercise or sports metabolomics; and studies that did not report interpretable metabolite or small-molecule responses. Studies limited to disease, aging, pharmacology, or preclinical models were excluded from the core evidence base unless they provided clear mechanistic insight into exercise-responsive metabolic pathways. When retained, they were treated as indirect mechanistic context, not direct evidence for athlete monitoring, training prescription, recovery management, or performance support.

S4. Data Extraction, Evidence Weighting, and Readiness Coding

For each relevant study, we extracted the athlete and exposure context, sampling and analytical details, metabolite findings, and links to athlete-relevant outcomes. Table S2 lists the extraction domains used to assess analytical confidence, source-evidence directness, validation maturity, outcome linkage, and added value beyond established monitoring measures.

We evaluated evidence on two independent axes. Source-evidence directness was classified as Level 1 human evidence from sport-trained participants (trained recreational, competitive, or elite athletes), Level 2 controlled human exercise evidence, or Level 3 indirect mechanistic evidence. Within Level 1, training status, competitive level, sport discipline, sex, age, biological matrix, exposure, and outcome were extracted as transferability descriptors; trained recreational evidence was not treated as directly transferable to elite athletes. Validation maturity was classified as

discovery, analytical validation with independent replication, longitudinal validation, or implementation testing. These categories correspond to source-evidence directness in main-text Table 5 and validation maturity in main-text Table 1. Keeping the axes separate prevents direct athlete-monitoring claims from being inferred from general exercise or indirect mechanistic evidence alone. Within-athlete longitudinal validation additionally requires context-matched repeated sampling, explicit consideration of analytical and biological variation, and separation of reliability under stable conditions from responsiveness to a defined exposure.

The framework separates mechanistically informative signals from candidate athlete-monitoring tools and specifies the conclusion permitted at each validation stage. A feature or panel was not regarded as ready for application simply because it differed between groups, changed before and after exercise, or was observed in athletes. Analytical measurability and athlete relevance alone do not establish longitudinal validity, added value, or implementation readiness.

The readiness pathway adapts, rather than substitutes for, established biomarker-development and qualification concepts. The BEST Resource and FDA qualification framework anchor interpretation in a defined context of use, while phased models distinguish discovery from analytical validation, longitudinal evaluation, and utility testing. The sport-specific contribution is to assess validation maturity separately from source-evidence directness and to require that longitudinal and implementation evidence be context matched to athletes, their sport, biological matrix, exposure, and monitoring question. It is an operational framework, not a regulatory qualification route or a source of universal decision thresholds.

Table S2 lists the extraction domains used to judge whether a study can inform athlete monitoring. Table S3 defines the four validation-maturity stages, their required evidence, and their permitted athlete-monitoring conclusions. These stages are distinct from the five operational roadmap steps in Section 6 of the main text; Step 3 specifies quantitative requirements within Stage 3, within-athlete longitudinal validation.

Table S2. Minimum extraction domains for athlete-monitoring interpretation

Domain	Minimum extracted information	Why it matters for athlete monitoring
Athlete and exposure context	Sport, training status (trained recreational, competitive, or elite), competitive level, exercise protocol, load, training phase, and intended use	Defines source-evidence directness and transferability; prevents extrapolation across incompatible training status, competitive level, or sport contexts
Sampling and biological context	Matrix, timing, recovery window, diet, hydration, sleep, travel, heat/altitude, menstrual-cycle information where relevant	Distinguishes acute flux, recovery, adaptation, and contextual confounding
Analytical confidence	Targeted or untargeted workflow, annotation/identification evidence, internal standards, pooled QC, batch effects, missingness, normalization, and precision	Prevents putatively annotated or analytically unstable features from being treated as reliable metabolites
Longitudinal and external validation	Repeated-measures design, within-athlete reliability, independent cohort replication, matrix/platform confirmation, and external validation	Tests whether a signal is stable and generalizable enough for candidate-panel development

Outcome linkage and added value	Predefined physiological, recovery, training-load, or performance outcome; comparison with established monitoring measures; practical sampling and turnaround	Determines whether a panel provides interpretable benefit beyond existing athlete-monitoring tools for a defined decision
Source-evidence directness and validation maturity	Source-evidence directness: Level 1 human evidence from sport-trained participants, reported as trained recreational, competitive, or elite; Level 2 controlled human exercise evidence; or Level 3 indirect mechanistic evidence; validation maturity: discovery, analytical validation with independent replication, longitudinal validation, or implementation testing	Keeps study-context directness distinct from validation maturity. Within Level 1, transferability depends on reported cohort characteristics and does not make recreationally trained findings directly applicable to elite athletes. Level 1 supports athlete-relevant interpretation and validation prioritization, but does not alone establish analytical validation, longitudinal validity, implementation utility, universal thresholds, or standalone decisions; see Table S3

Table S3. Four validation-maturity stages and permitted athlete-monitoring conclusions

Readiness stage	Minimum evidence	Permitted conclusion	Not permitted
Discovery	Transparent preprocessing, annotation confidence, missingness, batch assessment, and pooled QC	Hypothesis-generating feature or pathway signal	Individualized training or recovery decision, or a universal threshold
Analytical validation and independent replication	Chemical-confidence evidence, quantitative precision, relevant internal standards, and independent replication in a separate cohort and/or laboratory using a predefined protocol	Replicated candidate feature or panel requiring longitudinal validation	Generalizable threshold or application-ready panel
Within-athlete longitudinal validation	Context-matched repeated athlete samples; pre-specified baseline, exercise, and recovery windows; characterization of analytical variation and within-athlete biological variation under stable conditions; and responsiveness to a defined exposure. The number and timing of baseline samples must be justified for the specific analyte, matrix, population, and intended use.	Longitudinally supported candidate panel requiring implementation testing, when a context-interpretable trajectory and a prespecified association with an athlete-relevant outcome are shown. RCVs or individualized reference intervals may support interpretation only when their assumptions and underlying data are appropriate.	Universal baseline-sample number, RCV, individual reference interval, performance threshold, or routine implementation without external validation and added-value testing
Implementation testing	External validation, calibration and generalizability, practical feasibility, and comparison with established monitoring measures	Context-specific, application-ready panel for a defined monitoring decision only when added value is demonstrated	Autonomous decision-making or a universal prescription tool

Note: The five steps in the main-text evidence-readiness roadmap are operational design and evaluation steps, not additional validation-maturity stages. In particular, main-text Step 3 operationalizes the quantitative requirements of Stage 3 in this table.

S5. Narrative Synthesis Framework

We organized the synthesis around four sport-relevant domains: platform selection for defined sport-science questions; pathway-level interpretation and candidate-biomarker readiness; wearable biosensing, AI-assisted interpretation, and multi-omics integration; and implementation barriers and research priorities.

We distinguished biologically informative metabolite changes from candidate biomarkers that may warrant athlete-monitoring evaluation. Findings were interpreted in relation to exercise modality, sampling time, biological matrix, nutritional status, training background, recovery phase, analytical platform, and links to physiological or performance-related outcomes. No direction, magnitude, or recovery kinetic was treated as universal.

S6. Limitations of the Review Methodology

This review has methodological limitations. It is a narrative review informed by a structured search, rather than a formal systematic review or meta-analysis. The search did not include SPORTDiscus, and the original workflow did not retain a database-level record log; we therefore cannot provide reliable retrospective study-selection counts or claim exhaustive sport-science retrieval. The review also does not provide pooled effect estimates, formal risk-of-bias assessments, field-wide counts of validation maturity, or systematic study-level extraction of CVa, CVi, RCV, index-of-individuality, or baseline-sample estimates. These quantitative parameters are analyte-, assay-, matrix-, population-, and intended-use-specific; this review therefore provides operational reporting and validation requirements rather than universal numerical defaults. Exercise protocols, participant characteristics, sport disciplines, analytical platforms, sample matrices, and sampling times vary substantially, limiting direct comparison and transferability.

Evidence for wearable biochemical sensing, AI-assisted metabolomics, IMS-MS, and multi-omics integration is advancing rapidly, but it remains uneven. These areas were treated as hypothesis-generating unless studies provided validation in the intended trained, competitive, or elite population, repeated-measures designs, and links to functional or performance-related outcomes.
